# Long-term survival after pulmonary metastasectomy in patients with esophageal squamous cell carcinoma with lung metastasis

**DOI:** 10.1186/s13019-022-02017-z

**Published:** 2022-10-14

**Authors:** Chien-Ming Lo, Kai-Hao Chuang, Hsing-Hua Lai, Yu Chen, Li-Chun Chen, Hung-I Lu, Yen-Hao Chen, Shau-Hsuan Li

**Affiliations:** 1 Department of Thoracic & Cardiovascular Surgery, Kaohsiung Chang Gung Memorial Hospital and Chang Gung University College of Medicine, Kaohsiung, Taiwan; 2 Department of Hematology-Oncology, Kaohsiung Chang Gung Memorial Hospital and Chang Gung University College of Medicine, Kaohsiung, Taiwan

**Keywords:** Pulmonary metastasis, Esophageal squamous cell carcinoma, Video assisted thoracoscopic surgery, Metastasectomy

## Abstract

**Objectives:**

Esophageal squamous cell carcinoma with pulmonary metastasis has a poor prognosis, and the only treatment modality is systemic therapy such as chemotherapy. Previous studies showed that pulmonary metastasectomy may provide benefits and has been suggested in selected patients with colorectal cancer, renal cancer, and sarcoma. However, there were few literatures evaluating the impact and treatment outcome of pulmonary metastasectomy in esophageal squamous cell carcinoma patients with isolated lung metastases. Therefore, we conducted this study.

**Methods:**

We retrospectively reviewed our patients with esophageal squamous cell carcinoma with pulmonary metastasis. Patients with extrapulmonary metastasis were excluded. We categorized them into two groups - the pulmonary resection group and the systemic treatment only group. We compared the overall survival and progression-free survival between groups, and also analyzed the surgical modality, which includes single or multiple port surgery.

**Results:**

The analysis included 44 esophageal squamous cell carcinoma patients with lung metastasis. Among these 44 patients, 14 patients have received pulmonary metastasectomy, and 30 patients received systemic treatment only. Patients who received pulmonary metastasectomy had significantly better overall survival (*p* < 0.0001) and progression-free survival (*p* = 0.038) than those who received only systemic treatment. The one-year overall survival and progression-free survival were 100% and 48% in patients receiving pulmonary metastatectomy, and 49% and 33% in patients receiving only systemic treatment. Among 14 patients receiving pulmonary metastatectomy, 10 patients underwent single port surgery. There were no postoperative complications in these 14 patients.

**Conclusion:**

Esophageal squamous cell carcinoma patients with lung metastasis who can receive pulmonary metastasectomy have better prognosis, and some patients can achieve long-term survival. Our findings suggest that aggressive pulmonary metastasectomy is suggested in esophageal squamous cell carcinoma patients with if no contraindication.

**Visual Abstract:**

Key question: How about the role of pulmonary metastasectomy in esophageal squamous cell carcinoma patients with isolated lung metastasis?

**Key findings:**

Patients who received pulmonary metastasectomy had better overall survival and progression-free survival than those who received only systemic treatment.

**Take Home Message:**

Esophageal cancer with isolated pulmonary metastasis can be treated aggressively with pulmonary metastasectomy if no contraindication.

## Introduction

Esophageal cancer usually has a poor prognosis, especially in patients who develop pulmonary metastasis. However, some studies showed that pulmonary metastasectomy can benefit selected patients. [1–8] In other cancers, such as colon cancer, sarcoma or head and neck cancer, pulmonary metastasectomy may provide obvious survival benefit.[4] Yotsukura Masaya et al. published a study about head and neck cancer with pulmonary metastasis which also provide survival benefit. [9] Thoracoscopic surgery may also benefit these patients as it is associated with less complications and easier recovery after surgery. Single port thoracoscopic surgery is a well-developed approach; however, currently, literature is sparse on the benefits of metastasectomy in patients diagnosed with esophageal cancer with pulmonary metastasis. We hypothesized that pulmonary metastasectomy would benefit these patients and reviewed our surgical outcomes.

## Materials and methods

### Patient population

Patients diagnosed with esophageal cancer and pulmonary metastasis who received pulmonary metastasectomy from January 2014 to July 2020 at Kaohsiung Chang Gung Memorial Hospital, were reviewed retrospectively. We also reviewed the patients diagnosed with pulmonary metastasis without pulmonary resection in the same period and excluded those with extrapulmonary metastasis. All patients diagnosed with esophageal cancer were evaluated by a multidisciplinary team which included a thoracic surgeon, a medical oncologist, a radiation oncologist, a radiologist, and a gastroenterologist. Pre-treatment evaluation included the following procedures: a panendoscopy, contrast-enhanced chest computer tomography (CT), and endoscopic ultrasound and positron emission tomography/computed tomography (PET-CT) scan. The tumor node metastasis stage (TNM) was determined according to the 7th American Joint Committee on Cancer (AJCC) staging system. We excluded patients who refused treatment or were treated by radiotherapy alone. All patients received curative intent treatment, which included concurrent chemoradiotherapy and/or esophagectomy. This study was approved by the institutional review board of Chang Gung Memorial Hospital.

### Thoracoscopic surgery

The patients received thoracoscopic surgery from two different surgeons. All patients underwent double lumen intubation with one lung ventilation, while pulmonary metastasectomy was performed. We used the same operative room setting for all patients, including team members and surgical devices.

### Overall survival and progression free survival

These two outcomes were used to compare the results between single port surgery and multiple port surgery. We analyzed the result between the pulmonary metastasectomy group and systemic treatment group. Overall survival is defined as the period from initial diagnosis of pulmonary metastasis to the last contact date. If a patient was dead (regardless of the cause of death), we defined it as an event. If a patient was alive, we defined it as censored. Progression-free survival is defined as the period from initial diagnosis date of pulmonary metastasis to the date of disease progression or death. If the contrast-enhanced CT showed disease progression, we defined it as an event. If it showed stability or regression, we defined it as censored.

### Statistical analysis

Statistical analysis was performed with the MedCalc Statistical Software version 19.4.0 (MedCalc Software Ltd, Ostend, Belgium; https://www.medcalc.org; 2020). A χ2 test or Fisher’s exact test were used to compare data between the two groups. For survival outcomes, the Kaplan–Meier method was used for univariate analysis, and the difference between survival curves was analyzed by a log-rank test. In a forward fashion, parameters were entered into a Cox regression model to analyze their relative prognostic importance. For all analyses, two-sided tests of significance were used with p < 0.05 considered significant.

## Results

### Patient characteristics

The baseline characteristics of these 44 patients were described in Table 1. Among these 44 patients, 14 patients have received pulmonary metastasectomies, and the other 30 patients only received systemic therapy. The mean age of these 14 patients receiving pulmonary metastasectomies was 60.3 years, and the median age was 61 years (range, 47–68 years). There were no significant difference between two groups in age, tumor grade, primary tumor location, performance status, and lung metastasis number.

**Table 1:** Associations between pulmonary metastasis and clinicopathologic parameters in patients with esophageal cancer with and without resection

Parameters		Pulmonary metastasis		
		**Resection**	**Systemic** **treatment only**	**p-value**
Number		14	30	
Age		60.3 ± 6.2	59.4 ± 9.7	0.83
Clinical T classification	T1/2	0	3	0.23
	T3/4	14	27	
Clinical N classification	N0	2	2	0.42
	N positive	12	28	
Tumor grade	Grade 1	1	3	0.95
	Grade 2	11	23	
	Grade 3	2	4	
Primary tumor location	Cervical	3	0	0.017*
	Upper	2	12	
	Middle	7	12	
	Lower	2	10	
Performance Status	0	14	25	0.268
	1	0	4	
	2	0	1	
Lung Metastasis Number	1	8	8	0.352
	2	2	5	
	3	1	1	
	4	1	1	
	>=5	2	15	

T = the extent of the tumor, N = the extent of spread to the lymph nodes,*=Statistically significant. х^2^ test or Fisher’s exact test was used for statistical analysis

### Overall survival and progression free survival

**Fig. 1:**
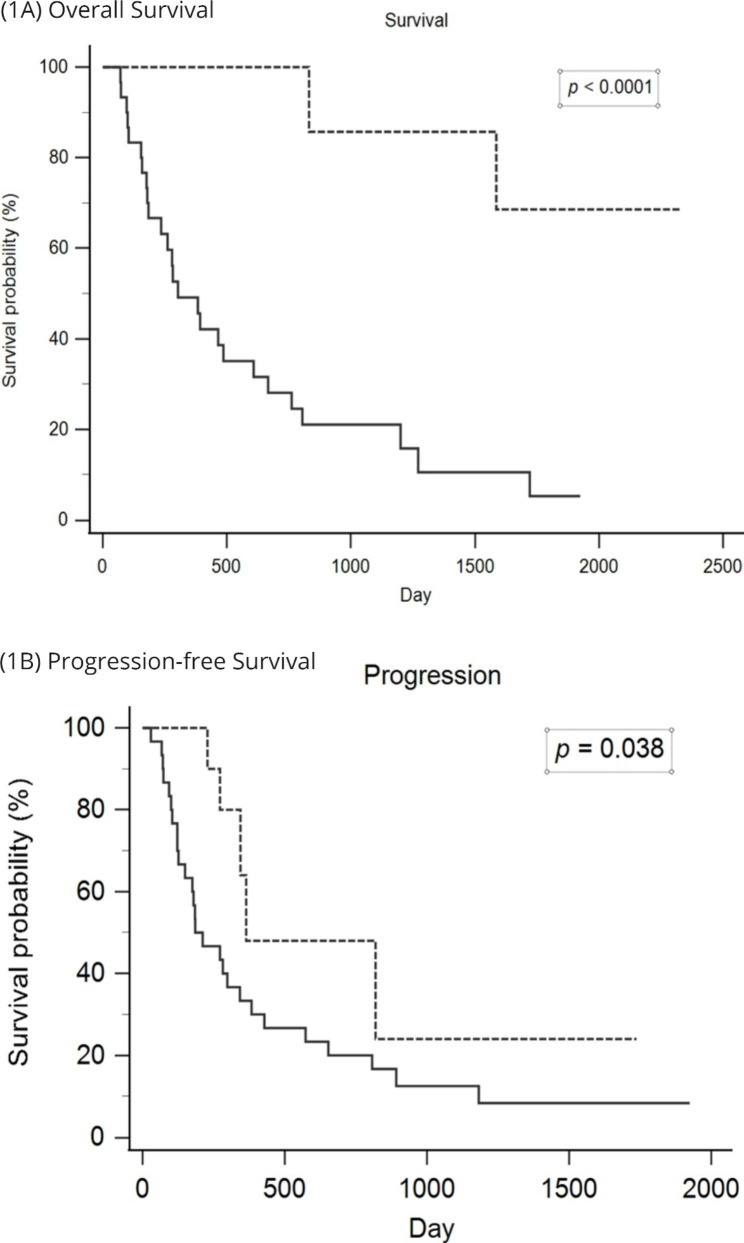
Pulmonary metastasectomy provides benefit in overall survival (**1 A**) and progression-free survival (**1B**) (Dotted line indicates pulmonary metastasectomy, solid line indicates systemic treatment)

The association between clinical parameters with overall survival and progression-free survival were shown in Table 2. Patients with better performance status have superior overall survival and progression-free survival. Tumor grade also has an impact in progression-free survival with p value of 0.019. Pulmonary metastasectomy (resection) has significant impact in both overall survival (Fig. 1 A) and progression-free survival (Fig. 1B) with p values of < 0.0001 and 0.038. One-year overall survival between pulmonary metastasectomy group and systemic treatment group is 100% and 49.1%. One year progression free survival between pulmonary metastasectomy group and systemic treatment group is 48.0% and 33.3%. Furthermore, we found that metastatic Tumor numbers did not have impact in overall survival and progression free survival.

**Table 2:** Results of univariate log-rank analysis of prognostic factors for overall survival and disease-free survival in patients diagnosed with esophageal cancer with pulmonary metastasis

Factors	No. of patients	Overall survival (OS)		Progression-free survival (DFS)	
		1-year OS rate (%)	p-value	1-year PFS rate (%)	p-value
Age					
> 60	19	67	0.43	45	0.93
≦ 60	25	62		35	
Clinical T classification					
T1/2	3	66	0.83	66%	0.46
T3/4	41	64		37%	
Clinical N classification					
N0	4	67	0.90	25%	0.60
N positive	40	64		42%	
Tumor grade					
Grade 1	4	100	0.18	75	0.019*
Grade 2	34	62		41	
Grade 3	6	50		0	
Primary tumor location					
Cervical	3	100	0.11	0	0.18
Upper	11	40		18	
Middle	18	71		49	
Lower	12	66		50	
Resection or Systemic Treatment					
Resection	14	100	< 0.0001*	48	0.038*
Only Systemic Treatment	30	49		33	
Performance Status					
0	39	70	< 0.0001*	45	0.0024*
1	4	25		0	
2	1	0		0	
Lung Metastasis Number					
1	16	75	0.061	35	0.6032
2	7	67		40	
3	2	100		100	
4	2	100		100	
>=5	17	44		34	

T = the extent of the tumor, N = the extent of spread to the lymph nodes,*=Statistically significant. х^2^ test or Fisher’s exact test was used for statistical analysis. *=Statistically significant

We also analyzed the multiple variables of survival with Cox proportional-hazards regression model in forward fashion. The result is listed in Table 3. The overall survival and progression-free survival decreased? by tumor grade of 2.86 (*p* = 0.0238) and 2.7968 (*p* = 0.0212). The overall survival increased with resection by 0.0781 (*p* = 0.0006). However, progression-free survival only decreased by resection of 0.3625 (*p* = 0.0402).

**Table 3:** Results of multiple variable analysis (Cox proportional-hazards regression) of prognostic factors for overall survival (a) and disease-free survival (b) in patients diagnosed with esophageal cancer with pulmonary metastasis

**(a) Overall Survival**					
Covariate	b	Std. Error	Exp(b)	95%CI of Exp(b)	*p* value
Tumor grade	1.0510	0.4651	2.8604	1.1495 to 7.1189	0.0238*
Resection or Systemic Treatment	-2.5500	0.7407	0.0781	0.0183 to 0.3335	0.0006*
**(b) Progression-free Survival**					
Covariate	b	Std. Error	Exp(b)	95%CI of Exp(b)	*p* value
Tumor grade	1.0285	0.4461	2.7968	1.1665 to 6.7054	0.0212*
Resection or Systemic Treatment	-1.0148	0.4946	0.3625	0.1375 to 0.9555	0.0402*

### Thoracoscopic surgery

A total of 15 thoracoscopic procedures were performed in these 14 patients receiving pulmonary metastasectomies. One patient received thoracoscopic surgery twice, as pulmonary recurrence occurred after the first surgery. We tried to compare multiple port thoracoscopic surgery with single port thoracoscopic surgery in these 15 procedures. Pre-operative tumor grade showed differences in the two groups (*p* = 0.03). Other parameters such as tumor pathology, clinical TNM classification, and primary tumor location did not show significant differences. Surgery-related parameters, such as post -operative hospital stay, subjective pain scores, complications such as pneumonia or wound infection, curative resection, and surgery before treatment, did not show significant differences in the two groups (see Table 4).

**Table 4:** Associations between thoracoscopic pulmonary metastasectomy and clinicopathologic parameters in 15 procedures

Parameters				Thoracoscopic metastasectomy		
			Single Port		Two or Three Port	p-value
Number			11		4	
Age (Mean ± Std. Deviation)			60.18 ± 6.4		62.25 ± 5.9	0.58
Tumor Pathology		SCC	10		4	0.55
		Adeno	1		0	
Clinical 7th AJCC stage		III	6		3	0.49
		IV	5		1	
Clinical T classification		T3	6		1	0.33
		T4b	5		3	
Clinical N classification		N0	4		0	0.17
		N1-3	7		4	
Clinical M classification		M0	6		3	0.49
		M1	5		1	
Treatment before diagnosis		Yes	5		2	0.88
		No	6		2	
Tumor grade		1	0		1	0.03*
		2	6		3	
		3	5		0	
Primary tumor location		Cervical	5		2	0.67
		Upper	1		0	
		Middle	3		2	
		Lower	2		0	
Post-operative hospital stay			7.91 ± 2.8		5.25 ± 1.3	0.09
Post-operative NRS		0	2		0	0.38
		1	3		0	
		2	4		2	
		3	2		2	
Post-operative complication			0		0	

*=Statistically significant. SCC = Squamous Cell Carcinoma, Adeno = Adenocarcinoma, AJCC = The American Joint Committee on Cancer, T = the extent of the tumor, N = the extent of spread to the lymph nodes, M = the presence of metastasis, NRS = Numeric Rating Scale for pain. Chi-square test, and independent t test was used for statistical analysis

## Discussion

In this study, we analyzed thoracoscopic pulmonary metastasectomy results in patients with esophageal cancer. Single or multiple port thoracoscopic pulmonary metastasectomy did not demonstrate differences in surgical outcomes and complications. Average hospital stay was a little shorter in multiple port surgery because some single port surgeries were performed bilaterally. We did not find any differences in patients’ subjective pain ratings as well.

Previous some studies have shown that pulmonary metastasectomy may has a survival benefit in selected patients in esophageal cancer. [1–3, 5, 6, 8].

However, some literatures showed that pulmonary metastasectomy performed in patients with multiple pulmonary metastasis, did not show benefit in patients with esophageal cancer[1, 10]. However, in our series, pulmonary resection demonstrated a significant benefit in overall survival and progression-free survival. The one-year overall survival and progression-free survival were 100% and 48% in patients receiving pulmonary metastatectomy, and 49% and 33% in patients receiving only systemic treatment. Although, there is a selection bias, some patients with esophageal cancer could achieve long term survival in aggressive pulmonary resection even though pulmonary metastasis developed. In our opinion, pulmonary metastasectomy may provide precise tissue diagnosis and critical information to direct early decision-making for further palliative treatment such as chemotherapy, which could prolong overall and progression-free survival. Besides, pulmonary metastasectomy may reduce tumor burden dramatically in selected patients which may also benefit the overall survival and progression-free survival. Previous studies[11–13] have shown that patients with larger tumor burden may have worse efficiency of chemotherapy.

There are several limitations in our study. The limited number of patients in the pulmonary metastasectomy group could lead to selection bias. Future studies can enroll patients from other medical centers to reduce this. Large database case-controlled study with propensity score match may have better evidence to conduct this conclusion of “pulmonary resection have benefit for lung metastasis in patients with esophageal cancer.”

In conclusion, some patients with esophageal cancer could achieve long term survival in aggressive pulmonary resection even though pulmonary metastasis developed.

## Data Availability

All data generated or analyzed during this study are included in this published article.
